# Reference genome of California walnut, *Juglans californica*, and resemblance with other genomes in the order Fagales

**DOI:** 10.1093/jhered/esad036

**Published:** 2023-06-19

**Authors:** Sorel Fitz-Gibbon, Alayna Mead, Scott O’Donnell, Zhi-Zhong Li, Merly Escalona, Eric Beraut, Samuel Sacco, Mohan P A Marimuthu, Oanh Nguyen, Victoria L Sork

**Affiliations:** Department of Ecology and Evolutionary Biology, University of California Los Angeles, Los Angeles, CA, United States; Department of Ecology and Evolutionary Biology, University of California Los Angeles, Los Angeles, CA, United States; Department of Ecology and Evolutionary Biology, University of California Los Angeles, Los Angeles, CA, United States; Department of Ecology and Evolutionary Biology, University of California Los Angeles, Los Angeles, CA, United States; Wuhan Botanical Garden, Chinese Academy of Sciences, Wuhan, China; Department of Biomolecular Engineering, University of California Santa Cruz, Santa Cruz, CA, United States; Department of Ecology and Evolutionary Biology, University of California, Santa Cruz, Santa Cruz, CA, United States; Department of Ecology and Evolutionary Biology, University of California, Santa Cruz, Santa Cruz, CA, United States; DNA Technologies and Expression Analysis Core Laboratory, Genome Center, University of California, Davis, CA, United States; DNA Technologies and Expression Analysis Core Laboratory, Genome Center, University of California, Davis, CA, United States; Department of Ecology and Evolutionary Biology, University of California Los Angeles, Los Angeles, CA, United States; Institute of the Environment and Sustainability, University of California Los Angeles, Los Angeles, CA, United States

**Keywords:** California black walnut, California Conservation Genomics Project, CCGP, Fagales, Juglandaceae, *Juglans californica*, *Juglans hindsii*, *Juglans* spp

## Abstract

*Juglans californica*, California walnut, is a vulnerable small tree that is locally abundant but restricted to woodland and chaparral habitats of Southern California threatened by urbanization and land use change. This species is the dominant species in a unique woodland ecosystem in California. It is one of 2 endemic California walnut species (family Juglandaceae). The other species, Northern California black walnut (*J. hindsii*), has been suggested controversially to be a variety of *J. californica*. Here, we report a new, chromosome-level assembly of *J. californica* as part of the California Conservation Genomics Project (CCGP). Consistent with the CCGP common methodology across ~150 genomes, we used Pacific Biosciences HiFi long reads and Omni-C chromatin-proximity sequencing technology to produce a de novo assembled genome. The assembly comprises 137 scaffolds spanning 551,065,703 bp, has a contig N50 of 30 Mb, a scaffold N50 of 37 Mb, and BUSCO complete score of 98.9%. Additionally, the mitochondrial genome has 701,569 bp. In addition, we compare this genome with other existing high-quality *Juglans* and *Quercus* genomes, which are in the same order (Fagales) and show relatively high synteny within the *Juglans* genomes. Future work will utilize the *J. californica* genome to determine its relationship with the Northern California walnut and assess the extent to which these 2 endemic trees might be at risk from fragmentation and/or climate warming.

## Introduction

California walnut (*Juglans californica* S. Watson, Juglandaceae) is a riparian woodland tree species of Southern California, sometimes within the coast live oak woodlands but also dominating its own woodland ecosystem (Rundel and Gustafson 2005). While naturally fragmented riparian woodlands do not have great area of coverage in Southern California, they play an important ecological role because they occur on the edge of the chaparral dry habitat and mesic woodland habitat, playing a role in stabilizing stream banks, helping water flow, and even acting as fire breaks (Rundel and Gustafson 2005). Moreover, they are also considered a delicacy by California Native Peoples (Anderson 2005). Under threat due to urbanization and fragmentation, California walnut is classified as nearly threatened on the IUCN Red List of Threatened Species (Stritch and Barstow 2018). Models of current and future land use combined with climatically suitable habitat predict that California walnut will lose about 54% of its habitat by 2080 as a result of human-caused habitat conversion (Riordan et al. 2015). The future of this species will be increasingly isolated population fragments facing warmer climates.

California walnut is one of 2 California endemic *Juglans* species, among the approximately 22 species of walnut worldwide, many of which are valued for their nut production, lumber quality, and use as root grafts for commercial species (McGranahan and Leslie 1991). The other California endemic is the Northern California black walnut, *J. hindsii* Jeps, which has molecular data support as a single and separate species (Fjellstrom and Parfitt 1994; Stone et al. 2009), rather than a variety of California walnut (*J. californica* var. *hindsii*) as previously thought (Hickman 1993). Both species belong to the black walnut section (*Juglans* sect. *Rhysocaryon*). The goal of this paper is to present the first chromosomal-level genome of *J. californica*. We also assess its genetic similarity with high-quality genomes from 3 other walnut species: *J. nigra* (sect. *Rhysocaryon*), *J. mandshurica* (sect. *Cardiocaryon*) (Ji et al. 2021), and *J. regia* Linn. (sect. *Juglans*) (Marrano et al. 2020; Ji et al. 2021). Previous work (Manos and Stone 2001; Aradhya et al. 2007) indicates that the 2 California species are more closely related with each other and *J. nigra* Linn. (all from the same section) than with the older and more distantly related walnut species, *J. manshurica* Maximowicz and *J. regia*, which evolved in Asia and this interpretation is validated with our results. We take advantage of the consistent methods of library preparation, sequencing, and assembly used by the California Conservation Genome Project (CCGP) across species to compare resulting genomes from both walnut and oak trees. In each case, the CCGP-constructed genomes are aligned to pre-existing high-quality reference genomes. We speculate that observed differences between the alignment statistics for the 2 genera may reflect differences in genome organization. This genome assembly will provide a foundational resource for future studies on the unique ecology, biogeography, evolutionary history, and conservation of California black walnuts.

## Methods

### Study species

*J. californica*, California black walnut, is an insect-pollinated, vertebrate-dispersed small winter deciduous tree that can reach a height of 15 m (Keeley 1990). The species occurs in the Southern California foothills of the inland South Coast Ranges, Transverse Ranges, and Peninsular Ranges, typically on north- and east-facing slopes with deep soils having a high water-holding capacity, though it also occurs in riparian areas (Keeley 1990). *J. californica* can be locally abundant within its restricted range, but it has a relatively limited distribution, whose range could extend across Southern California from inland to coastal regions, touching southward to San Diego County, eastward to the San Bernardino Mts. and northward to Santa Barbara County. *J. californica* is always the dominant tree species in walnut woodlands, such as those found in the southern Santa Inez, foothills of eastern Los Angeles County. Additionally, the natural distribution of *J. hindsii*, only recorded in Northern California, is threatened by landscape development and has an exceedingly limited range from the San Joaquin Valley and Sacramento Valley to the inner Coast Ranges of Northern California and San Francisco Bay Area. It has been listed in IUCN as a vulnerable species.

**Fig. 1.**
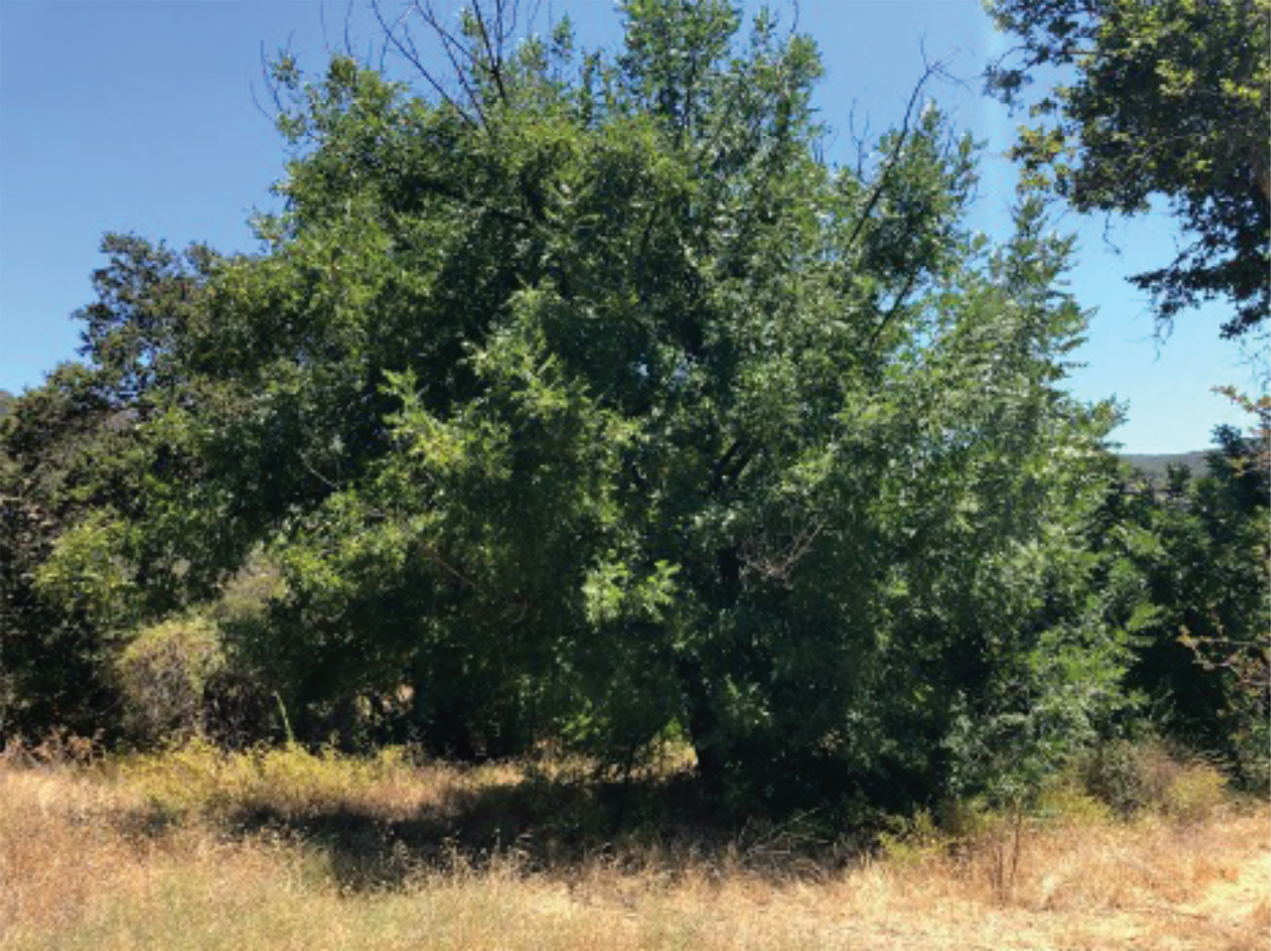
Photo of California black walnut (*Juglans californica*) located in a coastal chaparral/oak woodland habitat in the Santa Monica Mountains,UCLA Stunt Ranch Preserve, Calabasas, Los Angeles County, California, United States, a UC Natural Reserve System Ecological Preserve. Photo by Zhi-Zhong Li.

Leaf tissue from *J. californica* (GPS coordinates 34.09972, −118.6581) was sampled from an adult tree located at Stunt Ranch UC Reserve, Calabasas, California, United States on 25 July 2020 in coastal chaparral woodland habitat of the Santa Monica Mountains (Fig. 1). This adult tree was ~5 to 7 m tall with a single, well-defined trunk and full canopy.

### Nucleic acid extraction, library preparation, and sequencing

#### High molecular weight genomic DNA isolation.

High molecular weight (HMW) genomic DNA (gDNA) was extracted from 5 g of leaf tissue (Jcal-S-LA-6) using the Nanobind Plant Nuclei Big DNA Kit (Pacific Biosciences—PacBio, Menlo Park, California) and a plant-specific protocol (Workman et al. 2018) with the following modification. We used a nuclear isolation buffer supplemented with 350 mM Sorbitol to resuspend ground tissue and during the first wash of the nuclei pellet. The extracted HMW DNA was further purified using the high-salt-phenol-chloroform method (PacBio). The DNA purity (260/280 = 1.83 and 260/230 = 2.34) was assessed by absorbance ratios on a NanoDrop ND-1000 spectrophotometer. The DNA yield (47 ng/µL; 17 µg total) was quantified using QuantiFluor ONE dsDNA Dye assay (Promega, Madison, Wisconsin). The size distribution of the HMW DNA was estimated using the Femto Pulse system (Agilent, Santa Clara, California), and found that 51% of the total fragments were >90 kb.

#### HiFi library preparation and sequencing.

The HiFi SMRTbell library was constructed using the SMRTbell Express Template Prep Kit v2.0 (PacBio, Cat. #100-938-900) according to the manufacturer’s instructions. HMW gDNA was sheared to a target DNA size distribution between 15 and 20 kb. The sheared gDNA was concentrated using 0.45× of AMPure PB beads (PacBio, Cat. #100-265-900) for the removal of single-strand overhangs at 37 °C for 15 min, followed by further enzymatic steps of DNA damage repair at 37 °C for 30 min, end repair and A-tailing at 20 °C for 10 min and 65 °C for 30 min, ligation of overhang adapter v3 at 20 °C for 60 min and 65 °C for 10 min to inactivate the ligase, then nuclease treated at 37 °C for 1 h. The SMRTbell library was purified and concentrated with 0.45× Ampure PB beads (PacBio, Cat. #100-265-900) for size selection using the BluePippin system (Sage Science, Beverly, Massachusetts; Cat. #BLF7510) to collect fragments greater than 9 kb. The 15 to 20 kb average HiFi SMRTbell library was sequenced at University of California Davis DNA Technologies Core (Davis, California) using one 8 M SMRT cell, Sequel II sequencing chemistry 2.0, and 30-h movie on a PacBio Sequel II sequencer.

#### Omni-C library preparation and sequencing.

First, leaf tissue from Jcal-S-LA-6 is thoroughly ground with a mortar and pestle while cooled with liquid nitrogen. Nuclear isolation was performed using published methods (Workman et al. 2018) and the isolated nuclei were used as the starting input to the Dovetail Omni-C Kit (Dovetail Genomics, Scotts Valley, California) Mammalian Cells protocol. Briefly, chromatin was fixed in place in the nucleus. Fixed chromatin was digested under various conditions of DNase I until a suitable fragment length distribution of DNA molecules was obtained. Chromatin ends were repaired and ligated to a biotinylated bridge adapter followed by proximity ligation of adapter-containing ends. After proximity ligation, crosslinks were reversed and the DNA purified from proteins. Purified DNA was treated to remove biotin that was not internal to ligated fragments. An NGS library was generated using an NEB Ultra II DNA Library Prep kit (New England Biolabs—NEB, Ipswich, Massachusetts) with an Illumina compatible y-adaptor. Biotin-containing fragments were then captured using streptavidin beads. The post-capture product was split into 2 replicates prior to PCR enrichment to preserve library complexity with each replicate receiving unique dual indices. The library was sequenced at Vincent J. Coates Genomics Sequencing Lab (Berkeley, California) on an Illumina NovaSeq platform (Illumina, San Diego, California) to generate approximately 100 million 2 × 150 bp read pairs per GB of genome size.

#### Nuclear genome assembly.

We assembled the black walnut genome following the CCGP assembly protocol Version 2, outlined in Table 1, which uses PacBio HiFi reads and Omni-C data for the generation of high quality and highly contiguous nuclear genome assemblies. Briefly, we removed remnant adapter sequences from the PacBio HiFi dataset using HiFiAdapterFilt (Sim et al. 2022) and obtained the initial diploid assembly with the HiFi reads using HiFiasm (Cheng et al. 2021). The diploid assembly consists of 2 pseudo haplotypes (primary and alternate), where the primary assembly is more complete and consists of longer phased blocks, and the alternate consists of haplotigs (contigs with the same haplotype) in heterozygous regions, is not as complete and more fragmented. Given the characteristics of the latter, it cannot be considered on its own but as a complement of the primary assembly (https://lh3.github.io/2021/04/17/concepts-in-phased-assemblies, https://www.ncbi.nlm.nih.gov/grc/help/definitions/).

**Table 1.** Assembly pipeline and software used.

Assembly	Software and options^a^	Version
Filtering PacBio HiFi adapters	HiFiAdapterFilt	Commit 64d1c7b
K-mer counting	Meryl (*k* = 21)	1
Estimation of genome size and heterozygosity	GenomeScope	2
De novo assembly (contiging)	HiFiasm (--primary)	0.15-r327
Remove low coverage, duplicated contigs	purge_dups	1.2.6
Scaffolding		
Omni-C scaffolding	SALSA (-DNASE, -i 20, -p yes)	2
Gap closing	YAGCloser (-mins 2 -f 20 -mcc 2 -prt 0.25 -eft 0.2 -pld 0.2)	Commit 20e2769
Omni-C contact map generation		
Short-read alignment	BWA-MEM (-5SP)	0.7.17-r1188
SAM/BAM processing	samtools	1.11
SAM/BAM filtering	pairtools	0.3.0
Pairs indexing	pairix	0.3.7
Matrix generation	Cooler	0.8.10
Matrix balancing	HicExplorer (hicCorrectmatrix correct --filterThreshold -2 4)	3.6
Contact map visualization	HiGlass	2.1.11
	PretextMap	0.1.4
	PretextView	0.1.5
	PretextSnapshot	0.03
Organelle assembly		
Mitogenome assembly	MitoHiFi (-r, -p 50, -o 1)	Commit c06ed3e
Genome quality assessment		
Basic assembly metrics	QUAST (--est-ref-size)	5.0.2
Assembly completeness	BUSCO (-m geno, -l embryophyta)	5.0.0
	Merqury	2020-01-29
Contamination screening		
General contamination screening	BlobToolKit	2.3.3
Local sequence alignment	BLAST+	2.10

Software citations are listed in the text.

^a^Options detailed for non-default parameters.

We identified sequences corresponding to haplotypic duplications, contig overlaps, and repeats on the primary assembly with purge_dups (Guan et al. 2020) and transferred them to the alternate assembly. We scaffolded both assemblies using Omni-C data with SALSA (Ghurye et al. 2017, 2019). Using PacBio HiFi reads and YAGCloser (https://github.com/merlyescalona/yagcloser), we closed some of the remaining gaps generated during scaffolding.

To generate Omni-C contact maps for both assemblies, we aligned the Omni-C data against the corresponding assembly with BWA-MEM (Li 2013), identified ligation junctions, and generated Omni-C pairs using pairtools (Goloborodko et al. 2018). We generated a multiresolution Omni-C matrix with cooler (Abdennur and Mirny 2020) and balanced it with hicExplorer (Ramírez et al. 2018). We used HiGlass (Kerpedjiev et al. 2018) and the PretextSuite (https://github.com/wtsi-hpag/PretextView; https://github.com/wtsi-hpag/PretextMap; https://github.com/wtsi-hpag/PretextSnapshot) to visualize the contact maps. We checked the contact map from the primary assembly for major misassemblies. If found, we cut the assemblies at the gaps where misassemblies were found. No further joins were made after this step. We then checked for contamination using the BlobToolKit Framework (Challis et al. 2020). Finally, we trimmed remnants of sequence adaptors and mitochondrial contamination.

#### Mitochondrial genome assembly.

We assembled the mitochondrial genome of black walnut from the PacBio HiFi reads using the reference-guided pipeline MitoHiFi (https://github.com/marcelauliano/MitoHiFi) (Allio et al. 2020). The mitochondrial sequence of *Quercus acutissima* (NCBI:MZ636519.1) was used as the starting reference sequence. After completion of the nuclear genome, we searched for matches of the resulting mitochondrial assembly sequence in the nuclear genome assembly using BLAST+ (Camacho et al. 2009) and filtered out contigs and scaffolds from the nuclear genome with a percentage of sequence identity >99% and size smaller than the mitochondrial assembly sequence.

#### Genome size estimation and quality assessment.

We generated k-mer counts (*k* = 21) from the PacBio HiFi reads using meryl (https://github.com/marbl/meryl). The generated k-mer database was then used in GenomeScope (Ranallo-Benavidez et al. 2020) to estimate genome features including genome size, heterozygosity, and repeat content. To obtain general contiguity metrics we ran QUAST (Gurevich et al. 2013). To evaluate genome quality and completeness we used BUSCO (Manni et al. 2021) with the embryophyta ortholog database (embryophyta_odb10), which contains 1,614 genes. Assessment of base level accuracy (QV) and k-mer completeness was performed using the previously generated meryl database and merqury (Rhie et al. 2020). We further estimated genome assembly accuracy via BUSCO gene set frameshift analysis using the pipeline described in Korlach et al. (2017). Following data availability and quality metrics established in the Vertebrate Genome Project (VGP) pipeline (Rhie et al. 2021), we will use the derived genome quality notation *x*·*y*·*Q*·*C*, where *x* = log_10_[contig NG50]; *y* = log_10_[scaffold NG50]; *Q* = Phred base accuracy QV (quality value); *C* = % genome represented by the first “n” scaffolds, following a known karyotype of 2n = 32 (Woodworth 1930). Quality metrics for the notation will be calculated on the primary assembly.

#### Comparisons with other genomes.

The *J. californica* genome was compared with 3 publicly available *Juglans* reference genomes, *J. regia* (NCBI:GCF_001411555.2), *J. mandshurica* (NCBI:GCA_002916435.2), and *J. nigra* (NCBI:GCA_002916485.2). Nucleotide identity was determined from chromosome to chromosome lastz (Harris 2007) alignments using default (sensitive) parameters. To minimize error from poorly aligned regions and focus on nucleotide identity in clearly cognate regions, the set of longest alignments covering approximately 25% of the reference genome (*J. regia*) were used for the calculation. Bedtools (Quinlan and Hall 2010) and perl scripts were used to collapse overlapping alignments and calculate mean nucleotide identity for overlapping fragments (no weighting). AnchorWave (Song et al. 2022) alignments were used to more sensitively detect synteny. AnchorWave alignments require gene model annotations for the reference genome. *J. regia* was chosen as the reference genome to which all others were compared since at time of writing, *J. regia* was the only *Juglans* genome at NCBI with annotated gene models. Minimap2 (Li 2018) was used to lift over CDS regions from the *J. regia* genome to each of the other genomes, followed by chromosome-to-chromosome alignment with AnchorWave proalign -R 1 -Q1. Numbers for positions matched, gaps and unaligned were determined using the python package maf-convert and samtools (Li et al. 2009) following procedures in the AnchorWave publication (Song et al. 2022).

For additional comparison, exactly the same procedures were used to compare 3 high-quality *Quercus* (oak) genomes to the *Q. lobata* Née reference genome (NCBI:GCA_001633185.5), *Q. aquifolioides* Rehder & E. H. Wilson (NCBI:GCA_019022515.1), *Q. engelmannii* Greene (CCGP, unpublished), and *Q. mongolica* Fischer ex Ledebour (NCBI:GCA_011696235.1). RepeatModeler-2.0.3 (Flynn et al. 2020) was used for each of the *Juglans* and *Quercus* genomes to identify repeats, including lineage-specific repeats. The resulting database for each genome was used with RepeatMasker-4.1.2-p1 (Smit et al. 2013) to annotate and summarize the repeats.

## Results

**Fig. 2.**
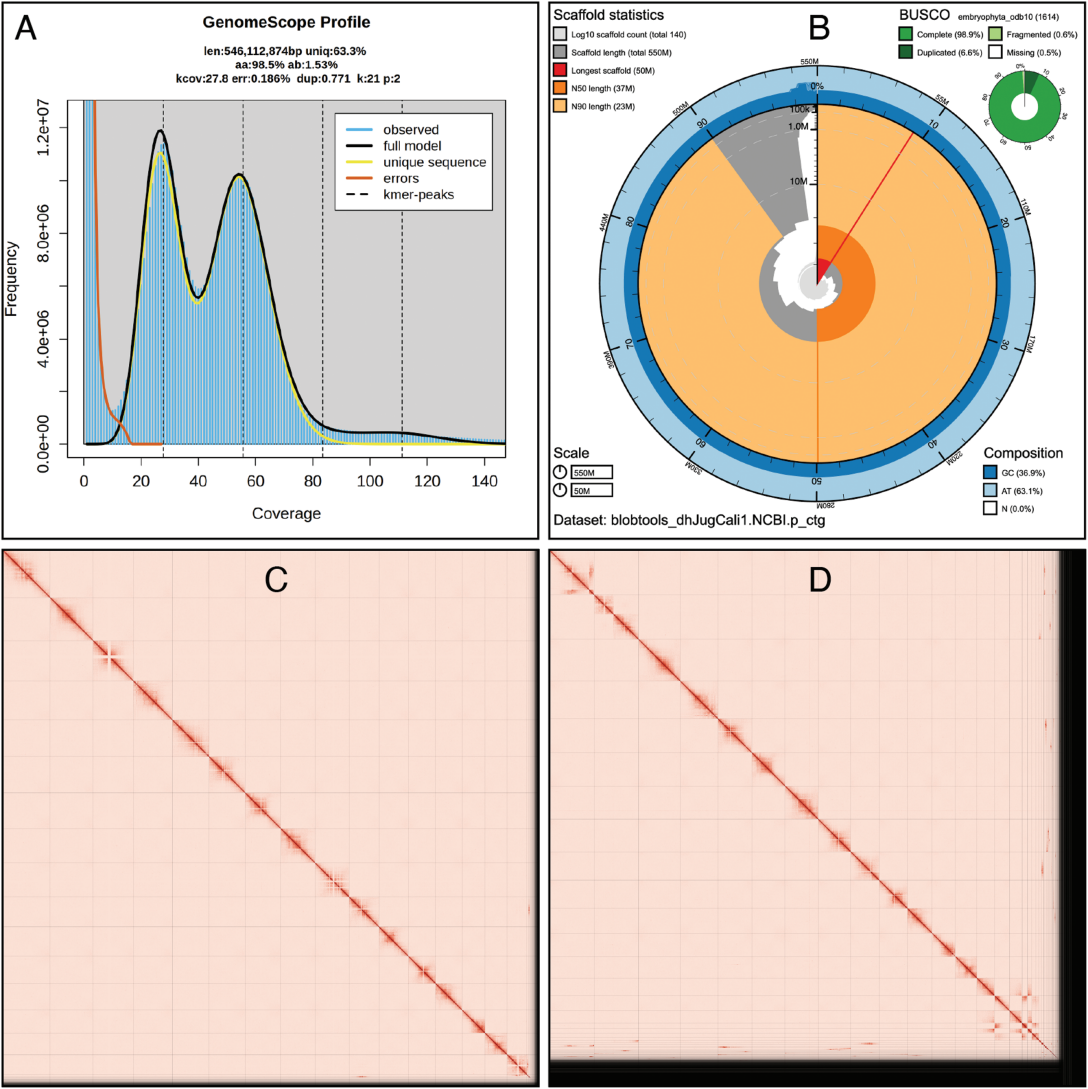
Visual overview of genome assembly metrics. A) K-mer spectra output generated from PacBio HiFi data without adapters using GenomeScope2.0. The bimodal pattern observed corresponds to a diploid genome and the k-mer profile matches that of high heterozygosity (>1.5%). K-mers covered at lower coverage and high frequency correspond to differences between haplotypes, whereas the higher coverage and slightly lower frequency correspond to the similarities between haplotypes. B) BlobToolKit Snail plot showing a graphical representation of the quality metrics presented in Table 2 for the *Juglans californica* primary assembly. The plot circle represents the full size of the assembly. From the inside out, the central plot covers length-related metrics. The red line represents the size of the longest scaffold; all other scaffolds are arranged in size-order moving clockwise around the plot and drawn in gray starting from the outside of the central plot. Dark and light orange arcs show the scaffold N50 and scaffold N90 values. The central light gray spiral shows the cumulative scaffold count with a white line at each order of magnitude. White regions in this area reflect the proportion of Ns in the assembly; the dark vs. light blue area around it shows mean, maximum and minimum GC vs. AT content at 0.1% intervals (Challis et al. 2020). Hi-C contact maps for the primary C) and alternate D) genome assembly generated with PretextSnapshot. Hi-C contact maps translate proximity of genomic regions in 3D space to contiguous linear organization. Each cell in the contact map corresponds to sequencing data supporting the linkage (or join) between 2 of such regions.

The Omni-C and PacBio HiFi sequencing libraries generated 63.6 million read pairs of Omni-C data and 1.9 million PacBio HiFi reads. The latter yielded 57.8-fold coverage (N50 read length 16,436 bp; minimum read length 47 bp; mean read length 16,253 bp; maximum read length of 48,288 bp) based on the GenomeScope2.0 genome size estimation of 546.1 Mb. Based on PacBio HiFi reads, we estimated 0.186% sequencing error rate and 1.54% nucleotide heterozygosity rate. The k-mer spectrum based on PacBio HiFi reads show (Fig. 2A) a bimodal distribution with 2 major peaks, at ~24- and ~56-fold coverage, where peaks correspond to homozygous and heterozygous states, respectively, of a diploid species. The distribution on the k-mer spectrum supports that of a high heterozygosity profile.

The final assembly (drJugCali1) consists of 2 pseudo haplotypes, primary and alternate, both genome sizes similar to the estimated value from GenomeScope2.0. The primary assembly consists of 138 scaffolds spanning 552 Mb with contig N50 of 30 Mb, scaffold N50 of 37 Mb, largest contig of 44 Mb, and largest scaffold of 50 Mb. On the other hand, the alternate assembly consists of 915 scaffolds, spanning 565 Mb with contig N50 of 25Mb, scaffold N50 of 34Mb, largest contig of 25 Mb, and largest scaffold of 48 Mb. Assembly statistics are reported in tabular and graphical form in Table 2 and Fig. 2B, respectively. The Omni-C contact maps suggest that the primary assembly is chromosome level and the alternate assembly, although not chromosome level, contains multiple chromosome-length scaffolds (Fig. 2C and D). The assembly process required little to no manual intervention. We closed 2 gaps from the alternate assembly and removed a total of 28 contigs from the assembly (7 from the primary, and 21 from the alternate) that satisfied the conditions for mitochondrial contamination.

**Table 2.** Sequencing and assembly statistics, and accession numbers.

BioProjects and Vouchers	CCGP NCBI BioProject			PRJNA720569			
	Genera NCBI BioProject			PRJNA765847			
	Species NCBI BioProject			PRJNA777184			
	NCBI BioSample			SAMN25062275			
	Specimen identification			Jcal.S.LA.6			
	NCBI Genome accessions			Primary	Alternate		
	Assembly accession			GCA_023349395.1	GCA_023349545.1		
	Genome sequences			JAKSXK000000000	JAKSXL000000000		
Genome Sequence	PacBio HiFi reads	Run		1 PACBIO_SMRT (Sequel II) run: 1.9 M spots, 31.6 G bases, 23.3 Gb			
		Accession		SRX15312200			
	Omni-C Illumina reads	Run		2 ILLUMINA (Illumina NovaSeq 6000) run: 63.6 M spots, 19.2 G bases, 6.2 Gb			
		Accession		SRX15312201, SRX15312202			
Genome Assembly Quality Metrics	Assembly identifier (quality code^a^)				dhJugCali1(7.7.Q64.C97)		
	HiFi read coverage^b^			57.88×			
				Primary	Alternate		
	Number of contigs			149	993		
	Contig N50 (bp)			30,178,554	7,285,237		
	Contig NG50 (bp)^b^			30,178,554	8,127,764		
	Longest contigs			43,958,334	25,558,138		
	Number of scaffolds			138	915		
	Scaffold N50 (bp)			36,519,750	34,496,276		
	Scaffold NG50 (bp)^b^			36,519,750	34,496,276		
	Largest scaffold			49,971,089	48,159,821		
	Size of final assembly (bp)			551,767,272	565,449,464		
	Gaps per Gbp (# Gaps)			5 (3)	136 (7)		
	Indel QV (frameshift)			47.97988761	48.24660866		
	Base pair QV			65.636	63.4494		
				Full assembly = 64.3934			
	k-mer completeness			81.1834	80.9444		
				Full assembly = 99.2245			
	BUSCO completeness (embryophyta) *n* = 1614		C	S	D	F	M
		P^c^	98.90%	92.30%	6.60%	0.60%	0.50%
		A^c^	98.90%	92.20%	6.70%	0.60%	0.50%
	Organelles		1 Partial mitochondrial sequence		JAKSXK010000138.1		

^a^Assembly quality code *x*·*y*·*Q*·*C* derived notation, from (Rhie et al. 2021). *x* = log_10_[contig NG50]; *y* = log_10_[scaffold NG50]; *Q* = Phred base accuracy QV (Quality value); *C* = % genome represented by the first “n” scaffolds, following a known karyotype of 2n = 32. Quality code for all the assembly denoted by primary assembly (dhJugCali.0.p). BUSCO Scores. (C)omplete and (S)ingle; (C)omplete and (D)uplicated; (F)ragmented and (M)issing BUSCO genes. *n*, number of BUSCO genes in the set/database.

^b^Read coverage and NGx statistics have been calculated based on the estimated genome size of 546.1 Mb.

^c^P(rimary) and (A)lternate assembly values.

**Fig. 3.**
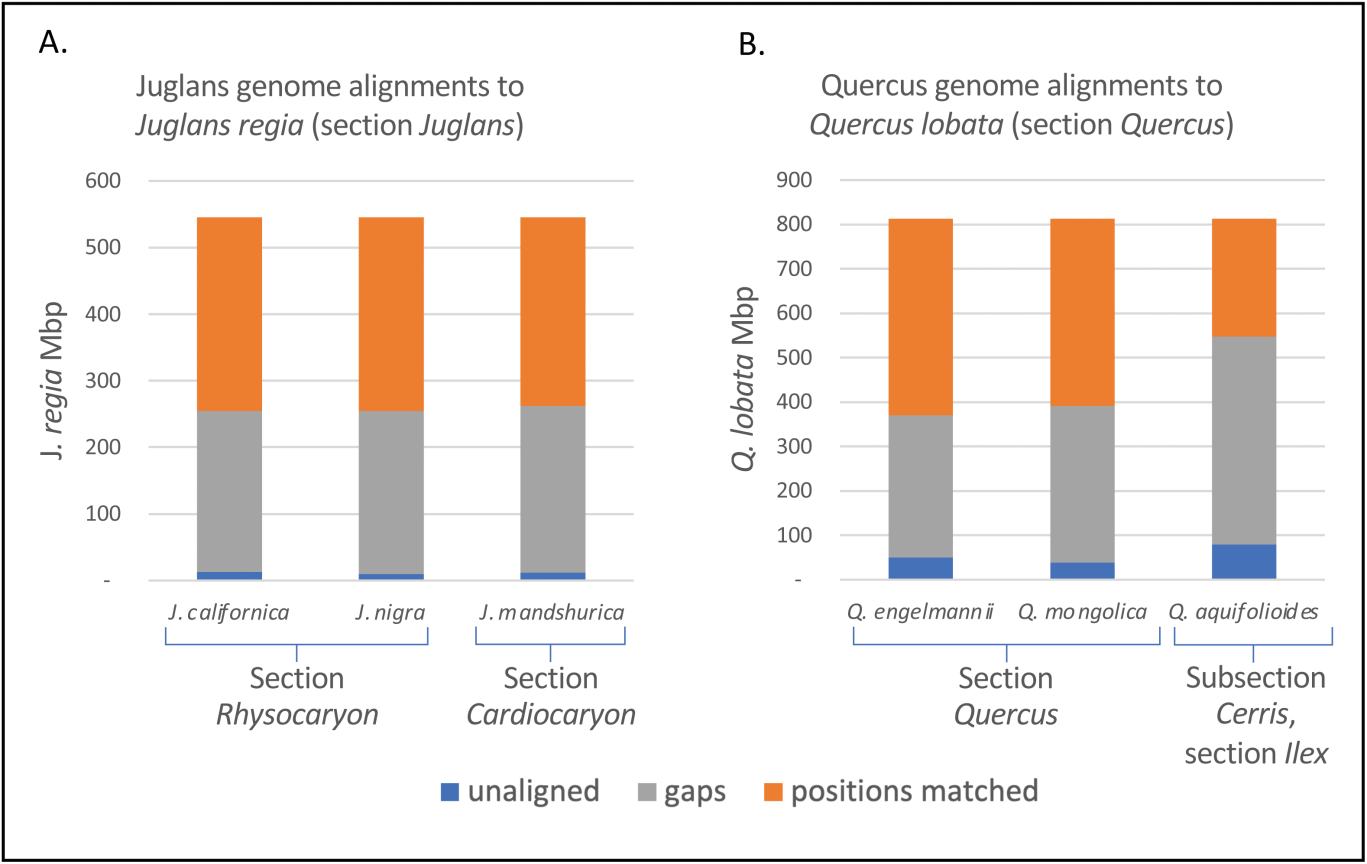
Proportion of genomes aligned by AnchorWave. Positions matched refer to nongap reference positions within syntenic alignments regardless of being a match or a mismatch. Gaps refer to gaps within syntenic blocks. Unaligned refers to the portion of the reference genome that does not occur in syntenic blocks. AnchorWave uses a sensitive method to find synteny in difficult to align regions and thus coverage is high, albeit with a high number of gaps. A) Alignment of *J. californica* and 2 other high-quality *Juglans* genomes to the *J. regia* reference genome. B) Alignment of 3 California oak genomes to the California Valley Oak, *Q. lobata*, reference genome.

**Fig. 4.**
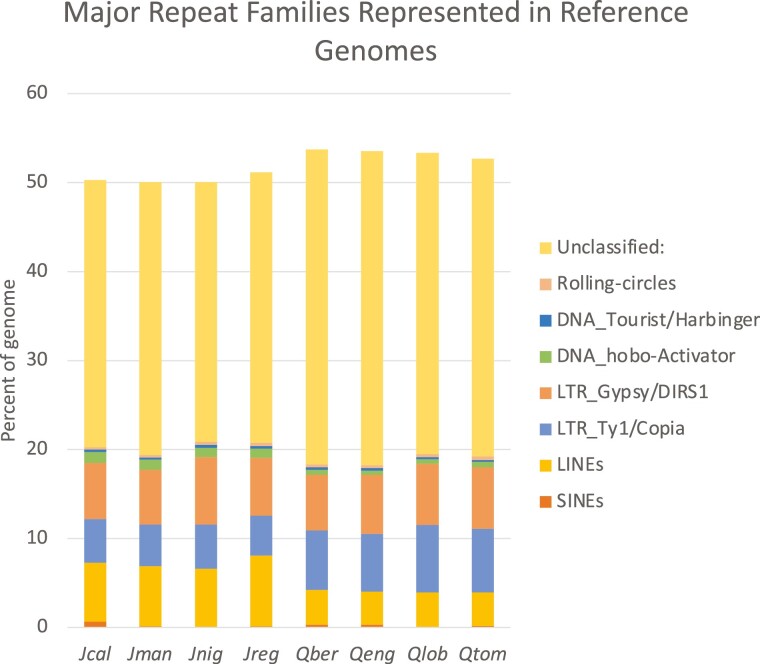
Comparison of the relative abundance of various major repeat families between genomes of *Juglans* [*J. californica* (*Jcal*), *J. manchurica* (*Jman*), *J. nigra* (*Jnig*), *J. regia* (*Jreg*)] and *Quercus* [*Q. berberidifolia* (Qber)*, Q. engelmannii* (*Qeng*)*, Q. lobata* (*Qlob), Q. tomentella* (*Qtom)*]. Repeats occupy a similar percentage of the genomes of both genera and have similar distributions of repeat types, albeit in both cases the majority of repeats are unclassified.

Comparisons of the *J. californica*, section *Rhysocaryon*, genome with 3 publicly available *Juglans* genomes: *J. nigra*, section *Rhysocaryon*, *J. regia*, section *Juglans*, and *J. mandshurica*, section *Cardiocaryon* show 98% identity for the 2 within section *Rhysocaryon*, and greater than 91% to 94% nucleotide percent identity between sections. Percent identities were measured over the longest alignments summing (after merging, see methods) to 25% of the reference genome (Table 3). AnchorWave was used to build highly sensitive coding-sequence-anchored alignments (Song et al. 2022), which yield larger contiguous alignments, spanning larger gaps. The results show that the vast majority of the *J. californica* genome aligns to the *J. regia* genome, with only approximately 2% of the *J. regia* genome unaligned (Fig. 3A). The same high coverage is seen for the other 2 *Juglans* genomes, which are also of high quality (Table 4). Of the aligned genomic positions, approximately half are position matched (i.e. a nongap aligned nucleotide, matched or mismatched) and half are gaps, which is to be expected for this type of alignment. Comparison to identically determined numbers for 3 *Quercus* (oak) species to the annotated *Q. lobata* genome (Fig. 3B) shows more variability and consistently higher numbers of unaligned positions in the oaks. The reason for this difference is unclear, but it does not seem to be due to the quality of the genome assemblies, since the *Quercus* genomes have consistently greater scaffold N50 lengths, and comparable contig N50 lengths (Table 3). Additionally, the 2 CCGP assembled genomes, *J. californica* and *Q. engelmannii*, which have substantially larger contig N50 lengths are not notably different from the others. Nor does the increased percent unaligned appear to be due to increased phylogenetic divergence as measured by section differences. Comparisons of the more completely aligned *Juglans* samples are all between sections, while 2 of the 3 *Quercus* comparisons are within sections (Fig. 3). Thus, it seems that *Juglans* genomes generally align more completely with each other than do *Quercus* genomes. The fraction of a genome that is repetitive can strongly affect genome assembly, even with long-read methods (Marx 2021), but this repetition does not explain the observed differences since *Juglans* and *Quercus* genomes have a similar array of repeat families present as similar fractions of their genomes (Fig. 4). Instead, differences in the distribution of repeats within the genomes is a possible explanation especially given that *Quercus* genomes have been shown to have numerous heterochromatic repeat regions spread throughout their chromosomes (Sork et al. 2022), a trait more often seen in grasses (Gouil and Baulcombe 2016; Sork et al. 2022). If so, the lower percentage of unaligned regions in the *Juglans* genomes would be an indication that they, like the majority of studied plant genomes, stably sequester most of their repeats in the pericentromeric regions of each chromosome.

**Table 3.**
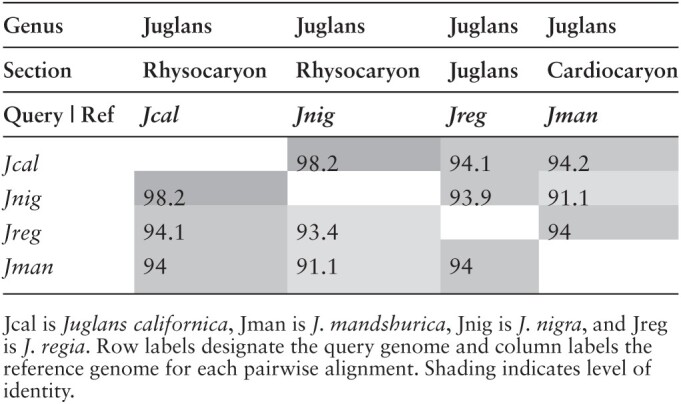
Nucleotide percent identity between 4 *Juglans* species based on the set of largest lastz alignments summing to 25% of the reference genome.

**Table 4.** Assembly metrics for genomes used for comparisons.

	Contig N50 (Mb)	Scaffold N50 (Mb)	Sequencing technology
Jreg	1.1	37	Illumina HiSeq; Oxford Nanopore MinION; Illumina NovaSeq
Jcal^a^	30	36	PacBio Sequel II; Dovetail
Jnig	2.4	35	Illumina HiSeq
Jman	1.4	36	Illumina HiSeq
Qlob	1	66	Illumina HiSeq; PacBio Sequel; Dovetail
Qeng^a^	35	57	PacBio Sequel II; Dovetail
Qmon	2.4	67	PacBio Sequel
Qaqu	1.4	64	PacBio RSII; Illumina

^a^Both *J. californica* and *Quercus engelmannii* were sequenced and assembled within the CCGP.

## Discussion

As expected, the 2 California walnut species within the same section (*Rhysocaryon*) aligned more closely with each other than with the 2 distantly related species, although all 4 species have relatively close alignments. We note our reported similarity comparisons among *Juglans* species are similar to those in a previous report (Stevens et al. 2018), although the magnitude of percent identities varies between the 2 studies due to different assessment methods. Specifically, our percent identities are higher as they are derived from only the top 25% longest alignments, thus avoiding difficult to align divergent regions. Whether the high identity across *Juglans* is due to a relatively recent evolutionary origin or a constraint on divergence is not known.

In general, *Juglans* genome structure appears to be more stably maintained than *Quercus* genomes, possibly related to the genus *Juglans* having only 17 species across 4 sections while *Quercus* has ~450 species across 2 subgenera and 8 sections. It is useful for walnut biologists that only 2% of walnut genomes fail to align to each other, even across sections when using a highly gap-tolerant alignment technique. In contrast to *Quercus* genomes where within-section alignments have 5% to 6% unaligned and a between-section alignment has close to 10% unaligned. Future work will look at the distribution of repeats across the *J. californica* genome in comparison to the *Q. lobata* genome, which has been shown to have numerous repeats throughout its chromosome arms rather than sequestering them in the pericentromeric regions (Sork et al. 2022).

Further work within the CCGP framework will include production and alignments of short-read whole genome sequence data from greater than 100 *J. californica* trees sampled from all areas of its California range, plus the same for *J. hindsii* across its California range. These data will allow a better understanding of the history and relationships between these 2 species. These data will be used in a landscape genomic analysis of neutral and climate-associated SNPs to examine the extent to which landscape features and climate factors have shaped the evolutionary history of walnuts in California and their vulnerability to environmental impacts.

## Data Availability

Data generated for this study are available under NCBI BioProject PRJNA720569. Raw sequencing data for sample Jcal-S-LA-6 (NCBI BioSample SAMN25062275) are deposited in the NCBI Short Read Archive (SRA) under SRX15312200-SRX15312202. Assembly scripts and other data for the analyses presented can be found at the following GitHub repository: www.github.com/ccgproject/ccgp_assembly.
